# A Hypoxia-Apoptosis Stress-Adaptation State Defines Immune-Low Melanoma and Predicts Metastatic Risk

**DOI:** 10.3390/cancers18121897

**Published:** 2026-06-10

**Authors:** Saige Yin, Dan Xu

**Affiliations:** 1 Department of Dermatology, The First Affiliated Hospital of Kunming Medical University, Kunming 650032, China; 2 Key Laboratory of Skin and Mucosal Injury Repair, Regeneration & Active Peptides of Yunnan Provincial Department of Education, Regenerative Medicine Research Center, Faculty of Basic Medical Science, Kunming Medical University, Kunming 650500, China

**Keywords:** melanoma, hypoxia, apoptosis, stress adaptation, immune microenvironment, metastasis

## Abstract

**Background/Objectives:** Chronic microenvironment stress contributes to the progression of melanoma, yet practical tools for quantifying tumor stress adaptation remain limited. **Methods:** Using disulfiram as a system-level probe, we identified shared regulatory modules between disulfiram-related targets and melanoma-associated genes, which are integrated into pathways regulating apoptosis and hypoxia/oxidative stress. From these modules, we devised a stress adaptation score (BiScore) based on hallmark gene characteristics and assessed its prognostic significance in melanoma cohorts. **Results and Conclusions:** In the TCGA-SKCM dataset, a higher binuclear score correlated with poorer overall survival and remained an independent prognostic factor even after adjusting for clinical covariates. This observation was externally validated for distant metastasis-free survival rate in the GSE65904 cohort. Tumors with high binuclear expression consistently exhibited reduced immune infiltration and were weakly associated with mutation load, suggesting that the stress adaptation axis operates independently of gene mutation burden. Single-cell analyses demonstrated that stress-related signals were distributed across both malignant and stress-responsive microenvironmental compartments. In melanoma cell lines, hypoxia induces HIF-1α expression and shifts apoptosis-related proteins towards an anti-apoptotic state, and NAC attenuated CoCl_2_-induced ROS accumulation. In conclusion, these findings establish BiScore as a quantitative framework for capturing stress adaptation states linked to adverse outcomes in melanoma.

## Simple Summary

Melanoma cells are continuously exposed to hypoxic and oxidative stress, but practical methods for evaluating tumor stress adaptation remain limited. Using disulfiram as a systems-level probe, we developed BiScore to measure hypoxia/ROS-associated and apoptosis-attenuated stress adaptation in melanoma. High BiScore tumors were associated with worse survival outcomes and an immune-remodeled tumor microenvironment. Single-cell analyses further showed that the stress-related signals were distributed across both melanoma cells and stress-responsive microenvironmental compartments, including CoCl_2_-induced ROS accumulation and its attenuation by NAC. In melanoma cell experiments, hypoxia-mimetic stress induced increased HIF-1α and reduced apoptotic signaling, consistent with the stress-adaptation pattern captured by BiScore. These findings provide a systems-level perspective for understanding melanoma progression beyond conventional mutation-based models.

## 1. Introduction

Melanoma is one of the most aggressive skin cancers and is characterized by marked molecular heterogeneity and complex interactions with the tumor microenvironment [1]. Over the past decade, immune checkpoint inhibitors and targeted therapies have improved survival outcomes in some patients [2]. Nevertheless, considerable differences in clinical outcome still exist [3,4]. Even patients with the same clinicopathological stage can show markedly different prognoses, indicating that traditional staging systems cannot fully reflect the biological diversity of melanoma [2]. Therefore, identifying molecular features associated with tumor progression may improve risk stratification and support more precise clinical decision-making [5,6].

As the tumor progresses, melanoma cells are continuously exposed to microenvironmental stresses, including hypoxia, oxidative stress, and inflammation [1,7]. To survive under these hostile conditions, tumor cells activate adaptive programs that enhance cellular tolerance, metabolic flexibility, and stress resistance [8,9]. Hypoxia-inducible factors, particularly HIF-1α, coordinate transcriptional responses to oxygen deprivation [10,11], whereas reactive oxygen species (ROS) act as context-dependent mediators of redox signaling and stress adaptation [9,12]. Meanwhile, evasion of apoptosis allows tumor cells to escape programmed cell death and maintain survival under sustained stress [12]. Taken together, enhanced adaptation to oxidative stress, along with reduced apoptosis, may represent two complementary aspects of a broader stress-adaptation state in melanoma [12]. This state may support tumor cell survival and progression in hostile microenvironments [9].

Emerging evidence indicates that stress adaptation not only influences tumor growth but may also contribute to metastasis and immune evasion [13]. Hypoxia-driven signaling has been associated with epithelial–mesenchymal transition (EMT), metabolic reprogramming, and increased metastatic potential in multiple tumor types [14]. In addition, oxidative stress and hypoxia can reshape the tumor immune microenvironment by affecting immune cell infiltration and function [13,15]. Despite these observations, most previous studies have focused on individual signaling pathways or specific molecular regulators, and a systematic framework integrating multiple stress-related biological dimensions into an interpretable risk model remains limited. In melanoma, the relationships among tumor stress adaptation, metastatic progression, and immune microenvironmental remodeling have not been comprehensively characterized [16,17].

To reduce subjective bias in candidate pathway selection, we first applied a network pharmacology strategy to generate hypotheses at the systems level [18,19]. In many drug-repurposing studies, analyses begin with compounds that have well-defined anticancer mechanisms, which may bias downstream analyses toward previously characterized oncogenic pathways [19,20]. By contrast, small molecules with broad molecular interaction profiles may serve as exploratory entry points for identifying stress-related regulatory modules across multiple biological processes.

In contrast, small molecules with broad molecular interaction profiles may provide a more suitable entry point for exploratory network-level analyses. Disulfiram is a well-known small-molecule compound with a broad spectrum of molecular interactions [20,21]. Its reported targets are involved in multiple biological processes, including redox regulation, apoptosis, and inflammatory signaling. Previous studies have also suggested that disulfiram may exert anticancer effects by modulating cellular stress responses and metabolic pathways [22,23,24]. In this study, disulfiram was used as an analytical entry point for network pharmacology to reduce predefined pathway bias and facilitate the identification of regulatory modules potentially involved in melanoma stress adaptation. Importantly, the purpose of this analysis was not to evaluate disulfiram as a therapeutic agent in melanoma, but rather to use it as a systems-level probe to explore stress-related signaling networks.

Based on the candidate pathways identified through network analysis, we further selected representative stress-related biological dimensions and constructed a stress–adaptation bi-axis score (BiScore) integrating apoptosis-related signaling and hypoxia/ROS pathways. This scoring framework was designed to quantitatively capture the stress-adaptation state of melanoma tumors. We subsequently evaluated the prognostic stratification performance of BiScore in the TCGA-SKCM cohort and validated its predictive value in an independent GEO dataset. And the relationships among BiScore, immune microenvironmental features, single-cell stress-response patterns, and cell-based hypoxia/ROS-associated phenotypes were further investigated in the research.

Together, this study proposes an interpretable systems-level framework for characterizing melanoma stress adaptation by balancing hypoxia/ROS-associated signaling and apoptotic regulation. By linking tumor stress-adaptation states with clinical outcomes and microenvironmental features, our findings provide new system-level insights into melanoma progression and may contribute to improved molecular stratification of patients.

## 2. Materials and Methods

### 2.1. Study Design

**Figure 1:**
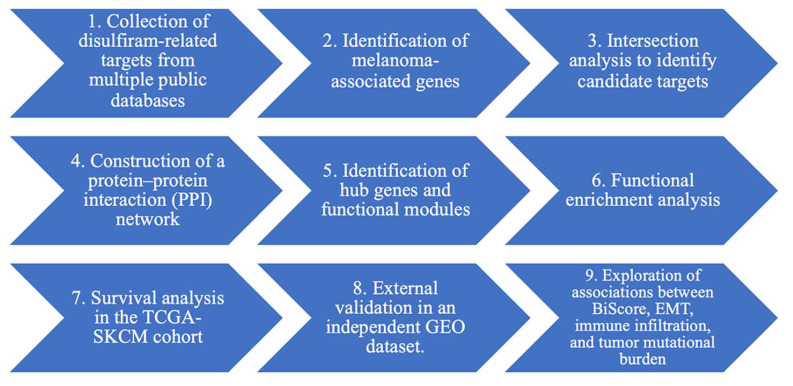
The analytical workflow for constructing a prognostic scoring model that reflects tumor stress–adaptation.

This study combined network pharmacology analysis with transcriptomic survival analysis to investigate potential molecular connections between disulfiram and melanoma. The analytical workflow included the following steps to construct a prognostic scoring model reflecting tumor stress–adaptation states (Figure 1): 

### 2.2. Identification of Disulfiram-Related Targets

Potential molecular targets associated with disulfiram were retrieved from several publicly available databases, including the Comparative Toxicogenomics Database (CTD, https://ctdbase.org/, accessed on 21 February 2026), GeneCards (https://www.genecards.org/, accessed on 21 February 2026), and SwissTargetPrediction (http://www.swisstargetprediction.ch/, accessed 21 February 2026). In CTD, chemical–gene associations with an inference score ≥35 were retained to reduce low-confidence interactions. In GeneCards, genes associated with disulfiram were retrieved using the keyword “disulfiram”, and genes with a relevance score ≥10 were retained. Predicted targets of disulfiram were additionally obtained from SwissTargetPrediction. To identify melanoma-associated genes, data were retrieved from CTD, GeneCards, and the Online Mendelian Inheritance in Man database (OMIM, https://www.omim.org/, accessed on 21 February 2026). In CTD, melanoma-related genes were filtered using an inference score ≥35, while in GeneCards genes associated with melanoma were obtained using the keyword “melanoma” and filtered with a relevance score ≥10. All melanoma-associated genes recorded in OMIM were also included. Gene lists obtained from all databases were integrated, duplicate entries were removed, and gene symbols were standardized according to the HUGO Gene Nomenclature Committee nomenclature to generate unified sets of disulfiram-related targets and melanoma-associated genes for subsequent analyses.

### 2.3. Identification of Intersecting Genes

To identify candidate genes potentially linking disulfiram and melanoma, an intersection analysis was performed between the disulfiram target set and the melanoma-associated gene set. The overlapping genes were considered candidate targets and were used for subsequent network construction and functional analyses.

### 2.4. Protein–Protein Interaction Network Construction

The intersecting genes were imported into the STRING database (version 11.5; https://string-db.org/, accessed on 21 February 2026) to construct a protein–protein interaction (PPI) network, restricting the analysis to Homo sapiens. A high-confidence interaction score (≥0.7) was used to filter reliable protein interactions. The resulting network was visualized and analyzed in Cytoscape (version 3.8).

### 2.5. Hub Gene Identification and Module Analysis

The PPI network was analyzed using the cytoHubba plugin in Cytoscape to identify key regulatory genes within. Nodes were ranked using the maximal clique centrality (MCC) algorithm, which has been reported to perform well in identifying essential nodes in biological networks. To further detect densely connected functional clusters, module analysis was performed using the MCODE algorithm implemented in the Metascape platform. Highly interconnected clusters were considered potential functional regulatory modules.

### 2.6. Functional Enrichment Analysis

Functional enrichment analysis was performed by using the Metascape platform (https://metascape.org/, accessed on 21 February 2026) to explore the biological functions of the intersecting genes. Gene Ontology (GO) biological process analysis and Kyoto Encyclopedia of Genes and Genomes (KEGG) pathway enrichment analysis were conducted to characterize the potential biological roles of these genes. Multiple testing correction was performed using the Benjamini–Hochberg method, and pathways with a false discovery rate (FDR) < 0.05 were considered statistically significant.

### 2.7. Construction of the Stress–Adaptation BiScore

To quantify tumor stress–adaptation states, a BiScore integrating apoptosis and hypoxia/oxidative stress signaling was constructed. Gene sets were obtained from the Molecular Signatures Database (MSigDB) Hallmark collection (https://www.gsea-msigdb.org/, accessed on 21 February 2026), including the Apoptosis, Hypoxia, and Reactive Oxygen Species pathways. Transcriptomic data from the TCGA-SKCM cohort were used to calculate pathway activity scores. Gene expression values were first standardized using Z-score normalization across samples, and for each signature, the pathway activity score was calculated as the average Z-score of genes within the corresponding gene set. Because increased apoptosis-related activity is generally associated with reduced tumor fitness and more favorable outcome, whereas enhanced hypoxia/ROS signaling was interpreted here as reflecting adaptive stress signaling, the apoptosis score was directionally inverted to ensure biological consistency when integrating the two axes into a single composite model. The final BiScore was calculated as BiScore = (−Apoptosis_Z + Hypoxia_ROS_Z)/2, and BiScore values were further standardized before downstream statistical analyses.

### 2.8. TCGA Cohort Survival Analysis

The TCGA Skin Cutaneous Melanoma (TCGA-SKCM) project via the UCSC Xena Browser (https://xenabrowser.net/) was used to obtained the transcriptomic data and corresponding clinical information for melanoma patients. Overall survival (OS) was defined as the primary endpoint, and progression-free interval (PFI) as the secondary endpoint. Patients were stratified by BiScore, and survival curves were generated using the Kaplan–Meier method; differences between groups were evaluated using the log-rank test. Cox proportional hazards regression models were constructed to assess the independent prognostic value of BiScore, and multivariable analyses, including age, sex, and AJCC stage, were performed while adjusting for available clinical covariates.

### 2.9. Time-Dependent ROC Analysis

The time-dependent receiver operating characteristic (ROC) analysis was used to evaluate the predictive performance of BiScore. The area under the ROC curve (AUC) was calculated at 1-, 3-, and 5-year time points to assess predictive accuracy. The concordance index (C-index) was calculated to assess the overall discriminative ability of the prognostic model.

### 2.10. External Validation

The melanoma dataset GSE65904, obtained from the Gene Expression Omnibus (GEO) database (https://www.ncbi.nlm.nih.gov/geo/), was used for external validation. Distant metastasis-free survival (DMFS) was defined as the primary endpoint, and disease-specific survival (DSS) as a secondary endpoint in the validation cohort. Kaplan–Meier survival analysis and Cox proportional hazards regression were performed using the same analytical procedures applied to the TCGA cohort.

### 2.11. EMT and Immune Infiltration Analysis

To explore the biological relevance of BiScore, the Hallmark EMT gene set from MSigDB was used to assess EMT activity. Immune and stromal infiltration levels were estimated using gene signature–based scoring approaches, including signatures representing CD8^+^ T cells, cytotoxic lymphocytes, natural killer cells, macrophages, dendritic cells, regulatory T cells, and other stromal components. Associations between BiScore and these signatures were evaluated using correlation analysis, and differences between risk groups were assessed using the Wilcoxon rank-sum test.

### 2.12. Tumor Mutational Burden Analysis

Somatic mutation data for TCGA-SKCM were obtained from the UCSC Xena somatic mutation dataset. Tumor mutational burden (TMB) was estimated based on the number of non-synonymous somatic variants per patient. The relationship between BiScore and TMB was assessed using correlation analysis, and survival analyses were additionally performed using combined stratification of BiScore and TMB.

### 2.13. Single-Cell Transcriptomic Analysis

Single-cell RNA-seq data from melanoma samples (GSE115978) were analyzed to explore the cellular distribution of BiScore-related stress programs within the tumor microenvironment. Standard Seurat-based workflows were applied for quality control, normalization, dimensionality reduction, clustering, and cell-type annotation. Cell populations, including malignant cells, T-cell subsets, macrophages, NK cells, B cells, cancer-associated fibroblasts (CAFs), and endothelial cells, were identified according to established marker genes. BiScore, Hypoxia/ROS, and Apoptosis module scores were calculated using gene signatures derived from the MSigDB Hallmark collection. Differences in module scores between cell populations were evaluated using the Wilcoxon rank-sum test. When multiple cell populations were compared, one-way ANOVA or the Kruskal–Wallis test was used as appropriate.

### 2.14. Cell Culture Conditions

Human melanoma cell lines A375 (CL-0014) and SK-MEL-28 (CL-0717) were obtained from Procell Life Science & Technology Co., Ltd. (Wuhan, China). All cells were cultured in Dulbecco’s modified Eagle’s medium (DMEM) containing 10% fetal bovine serum (FBS) and 100 U/mL penicillin-streptomycin in a 5% CO_2_ incubator at 37 °C.

### 2.15. Quantitative Real-Time Polymerase Chain Reaction (RT-qPCR)

Cells were seeded at a density of 3 × 10^5^/well in 6-well plates. After 24 h, the medium was changed to 0, 50, or 100 μM CoCl_2_ (Sigma-Aldrich, Saint Louis, MO, USA.) and the cultures were cultured for another 24 h. Total RNA was extracted from A375 and SK-MEL-1 cells using a Total RNA Extraction Kit (Beyotime, Shanghai, China). cDNA was synthesized using a Reverse Transcription Kit (TiansGen Biotech, Beijing, China), followed by amplification with an RT-qPCR amplification kit from the same manufacturer. Dissolution curve analysis confirmed single peak amplification. Relative gene expression levels were calculated using the 2^−^^ΔΔCT^ method, comparing the experimental groups to the control. Primer sequences were as follows: GAPDH: (f) CCAACGTGTCTGTTGTGGAT and (r) CTGCTTCACCACCTTCTTGA; HIF1-α: (f) AGCCGAGGAAGAACTATG and (r) TTTGATGGGTGAGGAATG; Bax: (f) GCCTCCTCTCCTACTTTG and (r) CTCAGCCCATCTTCTTCC; BCL-2: (f) TGGGAAGTTTCAAATCAGC and (r) GCATTCTTGGACGAGGG.

### 2.16. Western Blotting

After cells were lysed by the Radio Immunoprecipitation Assay Lysis (RIPA) buffer, the lysates were centrifuged at 14,000 rpm for 20 min at 4 °C, and supernatants were collected for protein quantification using the bicinchoninic acid (BCA) protein assay. Protein samples (30–50 μg) were resolved using 10–12% sodium dodecyl-sulfate polyacrylamide gel electrophoresis (SDS-PAGE) and transferred to 0.45-μm polyvinylidene fluoride membranes. Membranes were blocked with 5% skim milk powder (Solarbio, Beijing, China) at room temperature for 2 h, then incubated overnight at 4 °C with primary antibodies: β-actin and β-acin (Proteintech, Wuhan, China, 1:5000), HIF-1α (ABclonal, Wuhan, China, 1:4000), Bax (ABclonal, Wuhan, China, 1:500), and BCL-2 (ABclonal, Wuhan, China, 1:2000), and Cleaved-Caspase3 (Proteintech, Wuhan, China, 1:5000). After washing, membranes were incubated with goat anti-rabbit or anti-mouse secondary antibodies (Proteintech, Wuhan, China, 1:5000) for 1 h at room temperature. Protein bands were visualized using a chemiluminescence analyzer (Bio-Rad ChemiDoc™ XRS+ Imaging System, Hercules, CA, USA), and grayscale values were evaluated using ImageJ software (Version: 2.0.0-rc-69/1.52p).

### 2.17. ROS Activity Detection

Cells were seeded in the 6-well plates and divided into four groups: Control; CoCl_2_ (100 μM, 24 h); Acetylcysteine (NAC) (MCE, Shanghai, China, HY-B0215) (5 mM, 2 h) and CoCl_2_ + NAC (NAC pretreatment for 2 h before treated with 100 μM for 24 h). After 24 h, harvest the cells and use an ROS assay kit (Beyotime, Shanghai, China, S0033S) to measure ROS activity according to the manufacturer’s instructions. After DCFH-DA staining, fluorescence images were acquired under identical exposure conditions using a confocal laser microscope.

### 2.18. Statistical Analysis

All statistical analyses were performed using R software (version 4.5.2). Continuous variables were compared using the Wilcoxon rank-sum test, and correlations were assessed using Spearman’s correlation. The statistical tests were two-sided. One-way or two-way analysis of variance (ANOVA) and Kruskal–Wallis test were used for comparisons. and a statistically significant result was defined as *p* < 0.05.

## 3. Results

### 3.1. Apoptosis and Hypoxia/Oxidative Stress Were Identified as Core Stress Modules Linking Disulfiram and Melanoma by Network Analysis

**Figure 2:**
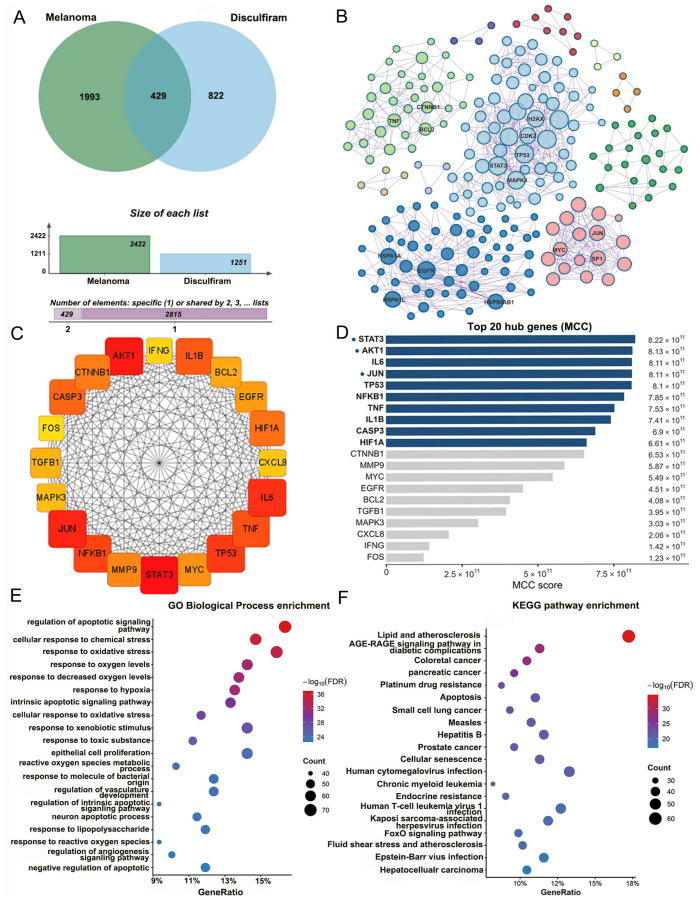
Shared targets between disulfiram and melanoma and network-based functional analysis. (**A**) Venn diagram of overlapping targets between disulfiram and melanoma. (**B**) Protein–protein interaction network and MCODE modules of the shared targets. (**C**) Circular visualization of the top hub genes identified by MCC analysis. (**D**) Ranking of the top 20 hub genes by MCC score. (**E**) GO biological process enrichment analysis. (**F**) KEGG pathway enrichment analysis.

To identify potential molecular intersections between disulfiram and melanoma, predicted disulfiram targets and melanoma-associated genes were integrated and analyzed for intersections. Venn analysis identified 429 overlapping genes, suggesting that disulfiram-related targets are broadly involved in melanoma-associated molecular networks (Figure 2A). A PPI network was subsequently constructed from these intersecting genes using the STRING database with a confidence score ≥0.7 (Figure 2B). The resulting network showed a highly interconnected topology, indicating close functional relationships among the candidate genes. Hub gene analysis was performed using the cytoHubba plugin in Cytoscape with the maximal clique centrality (MCC) algorithm, then identified the top 20 hub genes (Figure 2D), including key regulatory molecules such as STAT3, AKT1, TP53, NFKB1, TNF, IL6, CASP3, and HIF1A. These hub genes formed a central subnetwork with a prominent connectivity (Figure 2C). Functional enrichment analysis showed that the intersecting genes were mainly enriched in biological processes associated with the regulation of apoptotic signaling, oxidative stress response, and hypoxia response (Figure 2E). KEGG pathway analysis further showed enrichment in apoptosis, the FoxO signaling pathway, and cellular senescence (Figure 2F). Although inflammatory signaling pathways were also represented, the overall enrichment pattern suggested that apoptosis and hypoxia/oxidative stress were the dominant biological processes. To further examine the relationships among candidate stress-related dimensions, correlation analyses were performed in the TCGA-SKCM cohort (Figure S1). The inflammation axis showed a moderate correlation with the hypoxia/ROS axis, whereas the apoptosis axis showed weaker correlations with the other axes. Although the tri-axis model did not show obvious multicollinearity, its predictive performance was not better than that of the simplified bi-axis model. Therefore, taking both biological interpretability and statistical robustness into account, subsequent analyses were based on a bi-axis framework integrating apoptosis and hypoxia/ROS signaling.

### 3.2. Construction and Statistical Characteristics of the BiScore

**Figure 3:**
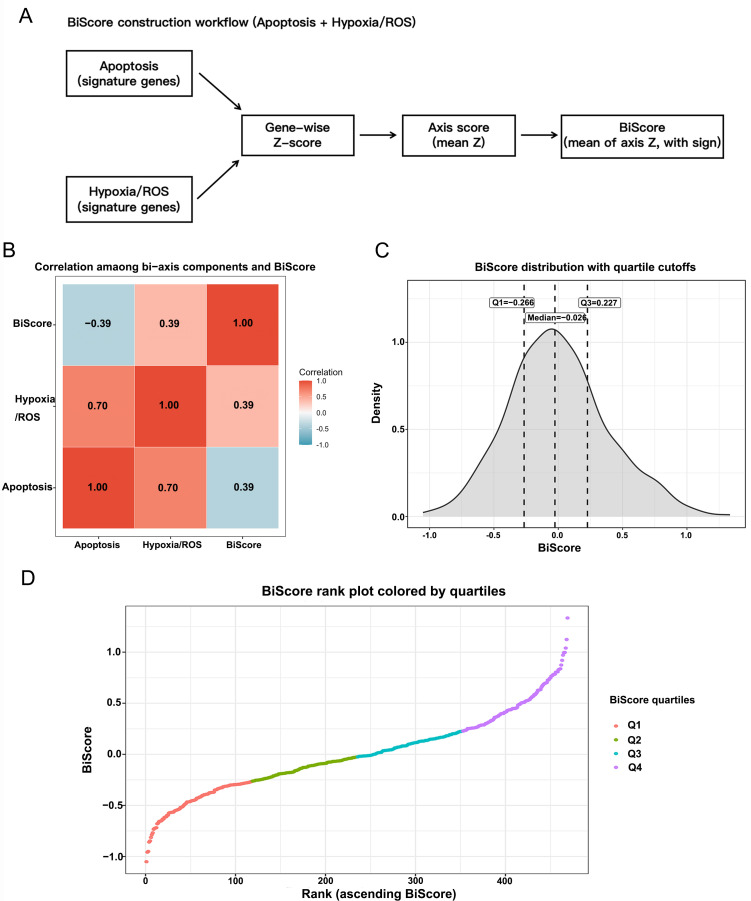
Construction and distribution of the BiScore in the TCGA-SKCM cohort. (**A**) Workflow for BiScore construction based on apoptosis and hypoxia/ROS signatures. (**B**) Correlations among the bi-axis components and BiScore. (**C**) Distribution of BiScore with quartile cutoffs. (**D**) Rank plot of BiScore colored by quartiles.

The workflow for BiScore construction is shown in Figure 3A. Gene expression values were first standardized using gene-wise Z-score normalization. The mean Z-scores for the Apoptosis and Hypoxia/ROS gene sets were then calculated to represent the activity of the two stress-related axes. Because increased apoptotic activity is generally associated with a better prognosis, whereas enhanced hypoxia/ROS adaptation reflects increased stress tolerance, the apoptosis score was sign-reversed prior to integration. BiScore was defined as the average of the two axis scores. Correlation analysis showed a moderate positive correlation between the Hypoxia/ROS axis and the Apoptosis axis (r = 0.70). In agreement with the model design, BiScore was positively correlated with the Hypoxia/ROS axis (r = 0.39) and negatively correlated with the Apoptosis axis (r = −0.39) (Figure 3B). BiScore values in the TCGA cohort exhibited a continuous, approximately symmetric distribution. The median was close to zero (median = −0.026), with clear quartile boundaries (Q1 = −0.266, Q3 = 0.227) (Figure 3C). Moreover, rank-order visualization showed a smooth gradient across samples without obvious discrete clustering, suggesting that BiScore may be used as a continuous risk variable in subsequent analyses (Figure 3D).

### 3.3. Independent Prognostic Value of BiScore in the TCGA Cohort

**Figure 4:**
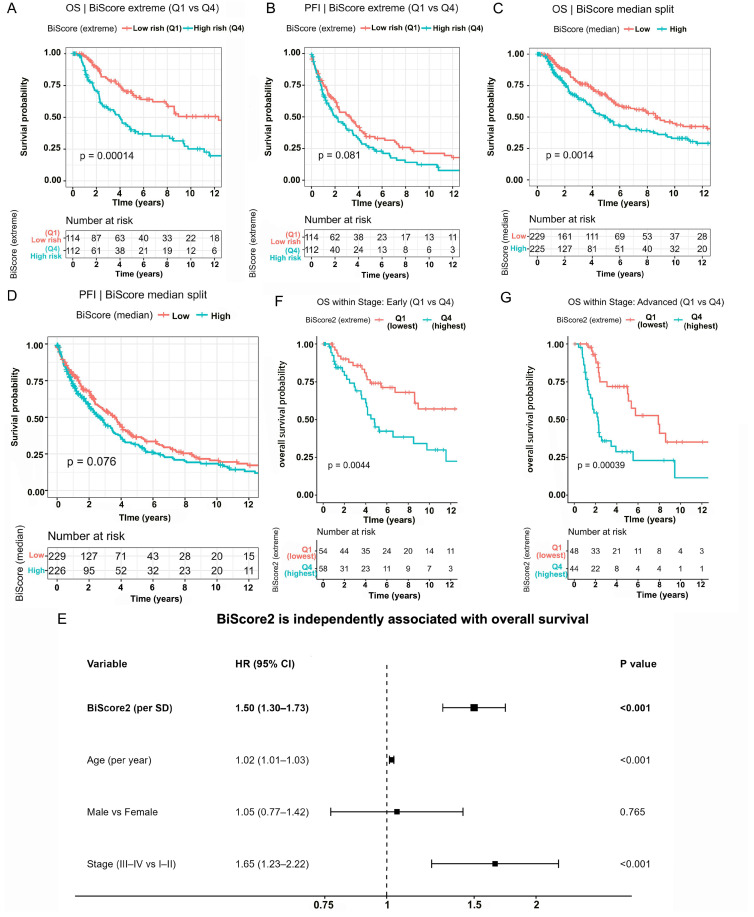
Prognostic value of BiScore in the TCGA-SKCM cohort. (**A**,**B**) Kaplan–Meier analyses of OS and PFI comparing extreme BiScore groups (Q1 vs. Q4). (**C**,**D**) Kaplan–Meier analyses of OS and PFI using median-based BiScore grouping. (**E**) Multivariable Cox regression analysis of OS including BiScore and clinical factors. The dashed line stands for 1, and bold information was for highlighting. (**F**,**G**) Kaplan–Meier analyses of OS in early-stage (I–II) and advanced-stage (III–IV) subgroups.

Kaplan–Meier survival analysis showed that BiScore was significantly associated with overall survival (OS). In the extreme-group comparison (Q1 vs. Q4), patients with high BiScore values had significantly reduced overall survival (Figure 4A, log-rank *p* = 0.00014). A similar trend was observed for progression-free interval (PFI), with a higher progression risk in the high BiScore group (Figure 4B, *p* = 0.081). Using a median-based grouping strategy, BiScore remained significantly associated with overall survival. Patients in the high-BiScore group showed significantly worse OS than those in the low-BiScore group (Figure 4C, log-rank *p* = 0.0014). For progression-free interval, the high-BiScore group also showed an unfavorable trend, although the difference did not reach statistical significance (Figure 4D, log-rank *p* = 0.076). These findings indicate that the prognostic signal of BiScore is preserved under a simpler dichotomization strategy, with a more robust effect on OS than on PFI. To evaluate whether BiScore provided prognostic information independent of clinical variables, multivariable Cox proportional hazards models were constructed that included age, sex, and AJCC stage (Figure 4E). BiScore remained significantly associated with overall survival. For each standard deviation increase in BiScore, the hazard ratio was 1.50 (95% CI 1.30–1.73, *p* < 0.001). Age (HR = 1.02, *p* < 0.001) and advanced stage (HR = 1.65, *p* < 0.001) were also significant risk factors, whereas sex was not statistically significant. The integrated model, including BiScore and clinical variables, achieved a concordance index (C-index) of 0.678, indicating moderate discriminative ability. Stratified analyses were further performed to assess the robustness of BiScore across clinical stages. In both early-stage (I–II) and advanced-stage (III–IV) subgroups, patients with higher BiScore values consistently had worse survival outcomes. In the extreme-group comparisons, the log-rank *p* values were 0.0044 in early-stage patients and 0.00039 in advanced-stage patients (Figure 4F,G). These findings suggest that the prognostic signal captured by BiScore was not simply a reflection of clinical stage but provided additional risk stratification across disease stages. Additional sensitivity analyses using follow-up truncation at 5000 and 6000 days showed that the overall direction of the PFI association remained consistent, supporting the robustness of the extreme-group stratification (Figure S2).

### 3.4. Predictive Performance of BiScore and Model Comparison

**Figure 5:**
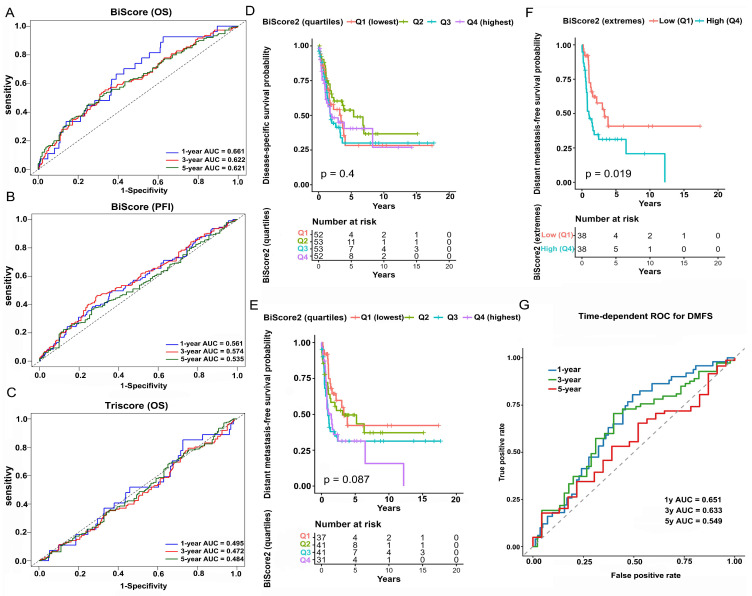
Time-dependent ROC analysis of BiScore and TriScore in the TCGA-SKCM cohort, and external validation of BiScore in the GSE65904 cohort. (**A**) Time-dependent ROC curves of BiScore for OS prediction. (**B**) Time-dependent ROC curves of BiScore for PFI prediction. (**C**) Time-dependent ROC curves of TriScore for OS prediction. (**D**,**E**) Kaplan–Meier analyses of DSS and DMFS across BiScore quartiles. (**F**) Kaplan–Meier analysis of DMFS comparing extreme BiScore groups (Q1 vs. Q4). (**G**) Time-dependent ROC analysis of BiScore for DMFS prediction.

Time-dependent ROC analysis was performed to evaluate the predictive performance of BiScore for overall survival. The area under the curve (AUC) values were 0.661 at 1 year, 0.622 at 3 years, and 0.621 at 5 years (Figure 5A). The relatively higher AUC at 1 year suggests that BiScore may provide more information for identifying early mortality risk. Although the AUC values declined slightly over longer follow-up periods, the model maintained stable predictive performance at around 0.62. For progression-free interval, the AUC values were 0.561, 0.574, and 0.535 at 1, 3, and 5 years, respectively (Figure 5B). Although the overall discriminative ability was limited, the overall risk direction remained consistent. In contrast, the previously evaluated tri-axis model (TriScore) showed markedly weaker predictive performance, with OS AUC values of 0.495, 0.472, and 0.484 at 1, 3, and 5 years, respectively (Figure 5C), which was close to random prediction. Therefore, subsequent analyses were based on the BiScore bi-axis model.

### 3.5. External Validation and Biological Associations of BiScore

External validation was performed using the independent GEO dataset GSE65904. Kaplan–Meier analysis showed that BiScore was not significantly associated with DSS in this cohort (Figure 5D, *p* = 0.4). In quartile-based analysis of distant metastasis-free survival (DMFS), no significant difference was observed among the four BiScore groups (Figure 5E, *p* = 0.367). However, BiScore was significantly associated with distant metastasis-free survival (DMFS), with the high BiScore group at higher risk of distant metastasis (Figure 5F, *p* = 0.019). Time-dependent ROC analysis further showed that the AUC values of BiScore for predicting DMFS were 0.651, 0.633, and 0.549 at 1, 3, and 5 years, respectively (Figure 5G).

**Figure 6:**
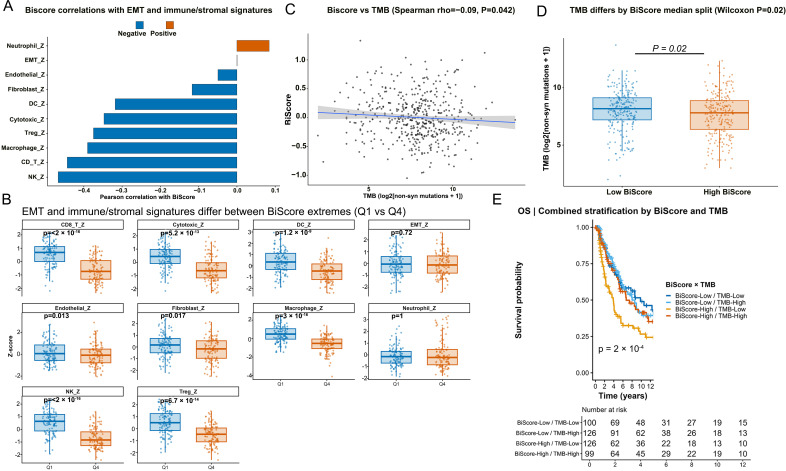
BiScore is associated with immune infiltration and tumor mutation burden in melanoma. (**A**) Correlations between BiScore and selected EMT and immune/stromal signatures in the TCGA-SKCM cohort. (**B**) Differences in selected EMT and immune/stromal signatures between the extreme BiScore groups (Q1 vs. Q4). (**C**) Correlation between BiScore and tumor mutation burden (TMB). (**D**) Comparison of TMB between low- and high-BiScore groups defined by median split. (**E**) Kaplan–Meier analysis of overall survival based on combined BiScore and TMB stratification. Time is shown in years.

To further examine the biological context represented by BiScore, associations with the tumor immune microenvironment were analyzed. Spearman correlation analysis showed consistent negative correlations between BiScore and multiple immune cell infiltration signatures, including CD8+ T cells, natural killer cells, cytotoxic lymphocytes, dendritic cells, and macrophages (Figure 6A). Moreover, extreme-group comparisons confirmed that tumors with higher BiScore values exhibited significantly reduced immune cell infiltration levels (Figure 6B). We also evaluated the relationship between BiScore and tumor mutational burden (TMB). Spearman correlation analysis demonstrated a weak negative correlation (ρ = −0.09, *p* = 0.042) (Figure 6C). Consistently, the high BiScore group showed a slightly lower TMB compared with the low BiScore group (Wilcoxon *p* = 0.02) (Figure 6D). A combined stratification analysis further revealed distinct prognostic patterns. The BiScore-high/TMB-low subgroup exhibited the worst survival outcomes, whereas the BiScore-low/TMB-high subgroup showed the most favorable prognosis (Figure 6E, log-rank *p* = 2 × 10^−4^). Collectively, these findings suggest that BiScore reflects a stress-adaptation state associated with a relatively immune-low tumor microenvironment remodeling.

### 3.6. Single-Cell Analyses Characterize the Stress-Adaptation Landscape Associated with Melanoma

**Figure 7:**
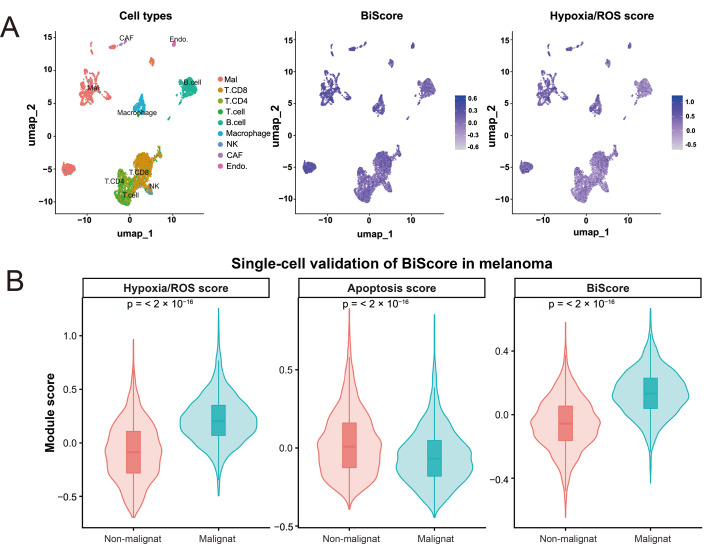
Single-cell validation of BiScore-related stress programs in melanoma. (**A**) UMAP plots showing that the major cell populations and the distribution of BiScore and Hypoxia/ROS scores across melanoma single-cell transcriptomes. (**B**) Violin plots showing higher Hypoxia/ROS score and BiScore but lower Apoptosis score in malignant cells compared with non-malignant cells. Statistical significance was determined by the Wilcoxon rank-sum test.

To further explore the cellular distribution of BiScore-related stress programs, single-cell RNA-seq data from melanoma samples (GSE115978) were analyzed. As shown in Figure 7A, the UMAP visualization identified distinct malignant melanoma cells (Mal) alongside multiple immune and stromal populations, including T-cell subsets, macrophages, CAFs, NK cells, B cells, and endothelial cells. Feature visualization showed that BiScore and Hypoxia/ROS-related signals were distributed across both malignant and selected stromal compartments, particularly endothelial and macrophage populations, consistent with the microenvironment-associated nature of hypoxic and oxidative-stress signaling. However, malignant cells overall exhibited greater stress-adaptation features than non-malignant populations (Figure 7A). Module-score analysis further showed that malignant cells had significantly higher Hypoxia/ROS scores and BiScore values, but lower Apoptosis scores, than non-malignant cells (Figure 7B). These findings suggested that malignant melanoma cells were enriched for a hypoxia/ROS-associated and apoptosis-attenuated stress-adaptation state. Collectively, these single-cell analyses support the existence of coordinated stress-adaptation programs involving both melanoma cells and stress-responsive microenvironmental compartments.

### 3.7. DepMap Analysis Supports the Functional Relevance of Representative BiScore-Axis Genes

To further evaluate whether representative components of the BiScore framework may contribute to melanoma cell fitness, DepMap CRISPR Chronos gene-effect scores were analyzed across melanoma cell lines. Representative genes were selected based on the biological architecture of the BiScore model, including hypoxia/ROS-associated genes (HIF1A, VEGFA, SLC2A1, LDHA, and BNIP3) derived from the Hallmark Hypoxia and ROS pathways, together with apoptosis-related genes (BCL2, BAX, and CASP3) from the Hallmark Apoptosis gene set. Several hypoxia/ROS-associated genes, particularly LDHA, SLC2A1, and BNIP3, exhibited relatively negative Chronos dependency scores across subsets of melanoma cell lines (Figure S3A), suggesting that components of the stress-adaptation program may contribute to melanoma cell fitness in a context-dependent manner. Heatmap visualization further demonstrated heterogeneous dependency landscapes across melanoma cell lines. In contrast, apoptosis-associated genes showed comparatively variable dependency patterns (Figure S3B), consistent with the dynamic and context-dependent nature of apoptotic signaling during stress adaptation. Importantly, these analyses were intended to provide orthogonal functional-context support for representative BiScore-axis genes rather than direct mechanistic validation.

### 3.8. Cell-Based Hypoxia-Mimetic Validation Supports the Directionality of the BiScore Bi-Axis Framework

**Figure 8:**
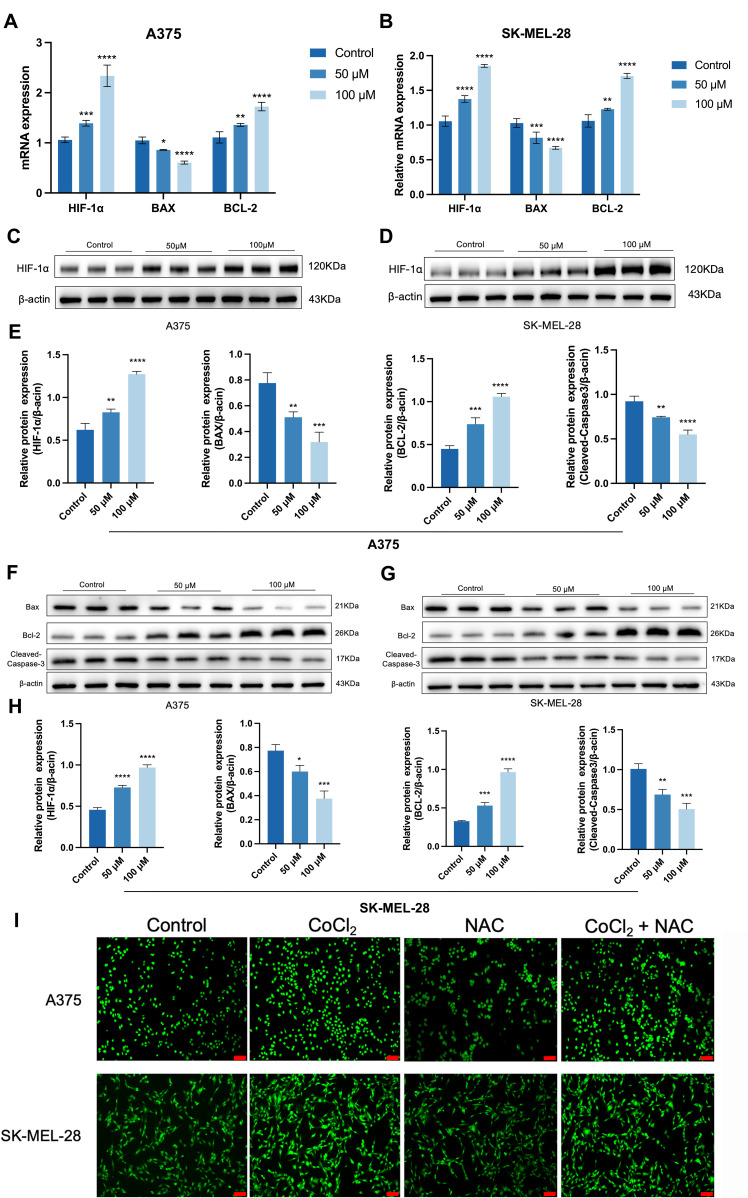
CoCl2 activates HIF-1α and attenuates apoptotic markers in melanoma cells. (**A**) mRNA expression of HIF-1α, Bax and Bcl-2 in A375 cells. (**B**) mRNA expression of HIF-1α, Bax and Bcl-2 in SK-MEL-28 cells. (**C**,**D**) Images of HIF-1α expression respectively in A375 and SK-MEL-28 cells. (**E**). Histogram of HIF-1α, Bax, Bcl-2, and cleaved caspase-3 protein expression in A375 cells. (**F**) and (**G**) Images of Bax, Bcl-2 and cleaved caspase-3 expression respectively in A375 and SK-MEL-28 cells. (**H**) Histogram of HIF-1α, Bax, Bcl-2, and cleaved caspase-3 protein expression in SK-MEL-28 cells. (**I**) Representative DCFH-DA fluorescence images of the intracellular ROS levels. bar (marked red) = 100 μm. *n* = 3. Data are means ± SEM. * *p* < 0.01, ** *p* < 0.05, *** *p* < 0.001, and **** *p* < 0.0001. The uncropped Western blotting images can be found in Supplementary Materials.

To establish the directionality of the hypoxic axis in the biaxial model, this study employed CoCl_2_, a commonly utilized chemical hypoxic simulation agent, to stably induce HIF-1α accumulation under normoxic culture conditions, thereby creating a controllable hypoxia-like stress state. It is important to note that chemical hypoxia does not fully replicate physiological hypoxia; hence, this experiment primarily aimed to verify the directional consistency of the hypoxic axis, rather than to simulate all biological processes associated with hypoxia. CoCl_2_ can elevate the mRNA levels of HIF-1α and BCL-2 in A375 and SK-MEL-28 cells, while reducing Bax mRNA levels (Figure 8A,B). In line with the mRNA level, this phenomenon was also observed at the protein level, and cleaved caspase-3 protein levels were reduced (Figure 8C–H). These results support the rationality of the hypoxia/apoptosis bi-axis framework we constructed. Intracellular ROS detection further showed that CoCl_2_ increased ROS accumulation in both melanoma cell lines, whereas NAC treatment attenuated CoCl_2_-induced ROS accumulation (Figure 8I). These findings support the involvement of ROS-related signaling in the CoCl_2_-induced hypoxia-mimetic stress state. Together, the cell-based results are consistent with the directional design of the BiScore framework, in which increased hypoxia/ROS-associated activity together with reduced apoptotic signaling reflects a stress-adaptive phenotype in melanoma cells.

## 4. Discussion

In this study, predicted disulfiram targets associated with melanoma-associated genes were integrated to construct a stress-adaptation network and, subsequently, BiScore, a bi-axis transcriptomic framework reflecting the balance between hypoxia/ROS-related stress adaptation and apoptotic signaling in melanoma. Score was significantly associated with prognosis, metastatic outcomes, and immune microenvironmental features (Figure 4, Figure 5 and Figure 6), suggesting that melanoma progression may depend not simply on oxidative stress intensity itself, but rather on the tumor’s ability to maintain a chronic adaptive equilibrium between hypoxia/ROS signaling and apoptotic regulation [25].

Network and enrichment analyses highlighted the oxidative stress response, hypoxia signaling, and regulation of apoptosis as the predominant stress-related processes captured by the intersecting disulfiram–melanoma network (Figure 2E,F). Several hub genes, including TP53, STAT3, AKT1, and HIF1A, have previously been implicated in stress adaptation and cell-fate regulation in cancer [26,27]. Importantly, disulfiram was used here not as a therapeutic intervention, but as a systems-level analytical probe to reduce predefined pathway bias and identify stress-adaptation–related regulatory modules relevant to melanoma progression.

Recent studies indicate that the relationship between ROS accumulation and apoptosis is highly context-dependent and cannot be interpreted as a simple linear process. Rather, cellular outcomes are shaped by the intensity and duration of oxidative stress, as well as by the cell’s intrinsic stress-response capacity [28]. Low-to-moderate and persistent ROS levels may promote adaptive signaling programs, including hypoxia-response activation, metabolic rewiring, mitochondrial homeostasis, and antioxidant defense, thereby supporting cell survival and phenotypic plasticity [28,29,30,31]. In contrast, excessive or sustained oxidative stress can disrupt mitochondrial function, cause loss of mitochondrial membrane potential, promote cytochrome c release, and activate caspase-dependent apoptotic pathways [28,32,33]. Thus, ROS may function as a threshold-dependent regulator of cell fate: within a tolerable range, ROS-related signaling can facilitate stress adaptation and survival, whereas beyond this threshold, it may trigger apoptotic cell death. In melanoma, this dual role is particularly relevant. On the one hand, ROS-associated transcriptional programs may help tumor cells maintain redox balance, metabolic flexibility, and adaptation to adverse microenvironmental conditions. On the other hand, uncontrolled oxidative stress can induce DNA damage, mitochondrial dysfunction, and apoptosis. Therefore, the BiScore model should not be interpreted as a direct measure of lethal ROS burden. Instead, it more likely reflects a chronic stress-adaptation transcriptional state characterized by enhanced hypoxia/ROS-associated signaling and relative suppression of apoptotic programs.

This interpretation is further supported by melanoma-specific mechanistic evidence linking ROS accumulation to both apoptotic signaling and immune sensitization. Sorrentino et al. showed that TRPM8 modulators induced ROS accumulation in melanoma cells together with mitochondrial membrane depolarization, cytochrome c release, and caspase-3 activation, indicating engagement of intrinsic mitochondrial apoptotic signaling. Importantly, the same study also demonstrated upregulation of the NK-cell activating ligand ULBP1 and enhanced melanoma susceptibility to NK-cell-mediated cytotoxicity through ULBP1–NKG2D engagement [34]. These findings provide direct melanoma-specific evidence that therapeutically induced ROS accumulation can be coupled to mitochondrial apoptosis and increased NK-cell immune susceptibility. Therefore, the immune consequences of ROS signaling in melanoma should be interpreted as context-dependent: chronic hypoxia/ROS-associated transcriptional programs may coexist with immune remodeling and apoptotic attenuation, whereas treatment-induced ROS accumulation may promote apoptosis-associated immune visibility through stress-ligand induction.

The cell-based validation further supported this directional interpretation. CoCl_2_-induced hypoxia-mimetic stress increased HIF-1α and BCL-2 expression while reducing BAX and cleaved caspase-3 levels in melanoma cells, indicating activation of hypoxia-associated stress programs together with attenuation of apoptotic execution signaling (Figure 8). In addition, CoCl_2_ increased intracellular ROS accumulation, whereas NAC treatment attenuated this increase, supporting the involvement of ROS-related signaling in the CoCl_2_-induced hypoxia-mimetic stress state. These findings provide experimental support for the hypothesis that the hypoxia-mimetic state captured in our model is accompanied by ROS accumulation and apoptosis attenuation, consistent with the biological directionality of the BiScore framework [34]. It should also be noted that disulfiram was not used here as a direct cellular stimulus because the purpose of the cell-based experiments was to validate the biological directionality of the BiScore framework under a controllable hypoxia/ROS-related stress condition, rather than to evaluate disulfiram itself as a functional effector in melanoma cells.

BiScore retained independent prognostic significance after adjustment for established clinical variables and remained informative across both early- and advanced-stage disease (Figure 4). External validation further supported its association with distant metastasis-related outcomes (Figure 5), suggesting that stress-adaptation signaling may contribute not only to survival advantage but also to metastatic progression during melanoma evolution [35]. Our immune analyses showed that high-BiScore tumors exhibited broad alterations in immune-cell infiltration patterns, including reduced CD8^+^ T-cell, NK-cell, dendritic-cell, and cytotoxic-lymphocyte signatures (Figure 6). Rather than representing a uniformly immune-suppressed state, these findings may reflect stress-associated immune remodeling during tumor progression. Prior studies have suggested that hypoxia and chronic stress adaptation can reshape the tumor immune microenvironment over time, promoting immune exclusion and altered immune-cell states compatible with tumor persistence [34,36,37]. Thus, the immune-low features observed in high-BiScore tumors may reflect coordinated tumor-intrinsic stress adaptation and microenvironmental remodeling rather than a single immune phenotype. This interpretation does not exclude the possibility that treatment-induced ROS accumulation may instead enhance melanoma immune visibility through stress-ligand induction and NK-cell-mediated cytotoxicity.

In addition, single-cell transcriptomic analyses showed that stress-related signals were represented not only in malignant melanoma cells but also in stress-responsive stromal compartments, including endothelial and macrophage populations (Figure 7), consistent with the multicellular ecosystem previously described in melanoma [38]. DepMap CRISPR analyses further demonstrated selective dependency patterns for representative BiScore-axis genes across melanoma cell line subsets (Figure S3), providing orthogonal functional-context support for the biological relevance of the framework [39]. Taken together, these analyses suggest that BiScore captures coordinated stress-adaptation states at the systems level, integrating tumor-intrinsic hypoxia/ROS signaling, apoptotic attenuation, and immune microenvironmental remodeling.

However, several limitations should also be acknowledged in this research. Firstly, although our cell-based assays support the directional relationship between hypoxia-mimetic stress, ROS accumulation, and apoptosis attenuation, the present study does not establish direct causal mechanisms linking individual BiScore-axis genes to melanoma progression. Secondly, while CoCl_2_ served as a useful hypoxia-mimetic model, it does not fully reflect the complexity of physiological hypoxia and ROS biology [40]. Finally, heterogeneity across external datasets may have influenced validation consistency. Future studies integrating physiological hypoxia models, spatial transcriptomics, and targeted perturbation experiments may further clarify how hypoxia/ROS signaling and apoptotic regulation jointly shape melanoma progression and immune remodeling.

## 5. Conclusions

In summary, we developed BiScore as a systems-level framework integrating hypoxia/ROS signaling and apoptotic regulation in melanoma. BiScore is associated with poor prognosis, increased metastasis risk, and remodeling of the immune microenvironment. Single-cell and DepMap analyses support the idea that this phenotype reflects a coordinated stress-adaptation network rather than a dependence on individual genes. CoCl_2_-induced hypoxia-mimetic experiments further validate the biological directionality of the framework. Together, these results suggest that melanoma progression depends on the tumor and microenvironment’s ability to maintain a chronic stress-adaptation state. BiScore provides a biologically grounded tool for understanding melanoma progression beyond conventional mutation- and clinicopathology-based stratification.

## Data Availability

The original contributions presented in this study are included in the article/Supplementary Materials. Further inquiries can be directed to the corresponding authors.

## Supplementary Materials

The following supporting information can be downloaded at: https://www.mdpi.com/article/10.3390/cancers18121897/s1, Figure S1. Collinearity and correlation diagnostics for axis selection. (A) Variance inflation factor (VIF) across all bi-axis pairs computed as VIF = 1/(1−r2), where r is the Pearson correlation between axis scores. (B) Correlation heatmap among the three axes (Apoptosis, Inflammation, Hypoxia/ROS) and the harmonized TriScore, illustrating the dependence structure used for model selection. Figure S2. Sensitivity analysis using truncated follow-up. Kaplan–Meier analyses of PFI comparing extreme BiScore groups (Q1 vs Q4) after truncating follow-up time at (A) 5000 days and (B) 6000 days. Time is displayed in years; log-rank P values and numbers at risk are shown. Figure S3. DepMap dependency of representative BiScore-axis genes. (A) Mean Chronos gene-effect scores of selected hypoxia/ROS- and apoptosis-related genes in melanoma cell lines. (B) Heatmap showing heterogeneous gene-effect patterns across melanoma cell lines. More negative scores indicate stronger dependency. File S1. The uncropped original Western blotting images.

## Abbreviations

The following abbreviations are used in this manuscript:

HIF	Hypoxia-inducible factors
ROS	Reactive Cxygen Species
EMT	epithelial–mesenchymal transition
BiScore	bi-axis score
PPI	protein–protein interaction
MCC	maximal clique centrality
GO	Gene Ontology
MSigDB	Molecular Signatures Database
OS	Overall survival
PFI	progression-free interval
GEO	Gene Expression Omnibus
DMFS	Distant metastasis-free survival
DSS	disease-specific survival
TMB	Tumor mutational burden
RT-qPCR	Quantitative real-time polymerase chain reaction
WB	Western Blotting
SDS-PAGE	sodium dodecyl-sulfate polyacrylamide gel electrophoresis
