# Supplementary material for: A Hypoxia-Apoptosis Stress-Adaptation State Defines Immune-Low Melanoma and Predicts Metastatic Risk
Source: Cancers (Basel). 2026 Jun 10;18(12):1897. doi: 10.3390/cancers18121897 (PMC13297633; doi:10.3390/cancers18121897)
Supplement: Supplementary file 1 [file cancers-18-01897-s001.zip › File S1. The uncropped original Western blotting images..pdf]

Figure 8C

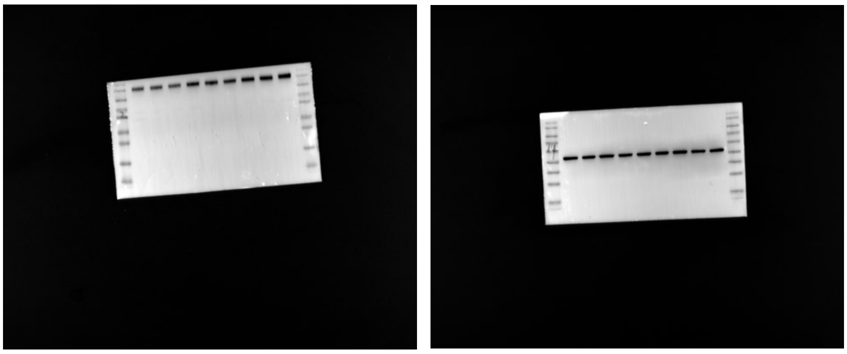

HIF-1a β-actin

Figure 8D

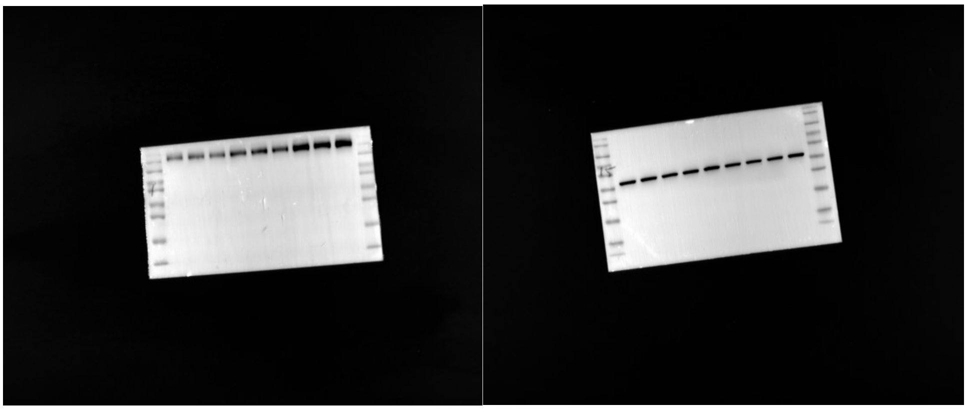

HIF-1a β-actin

Figure 8F

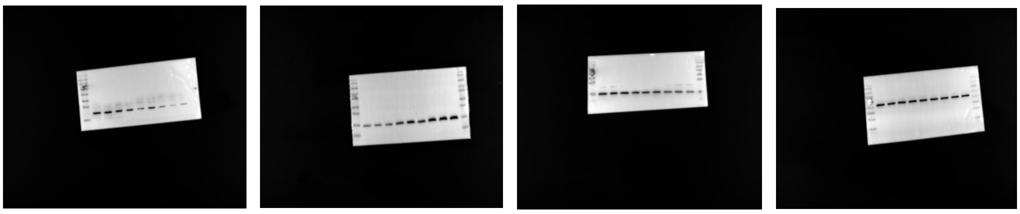

Bax BCL-2 cl-caspase3. β-actin

Figure 8G

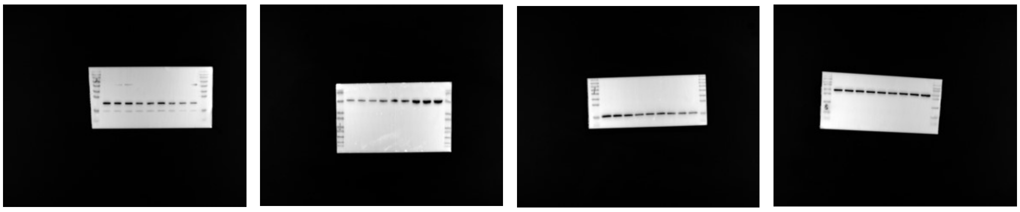

Bax BCL-2 cl-caspase3. β-actin
