# Supplementary material for: A Hypoxia-Apoptosis Stress-Adaptation State Defines Immune-Low Melanoma and Predicts Metastatic Risk
Source: Cancers (Basel). 2026 Jun 10;18(12):1897. doi: 10.3390/cancers18121897 (PMC13297633; doi:10.3390/cancers18121897)
Supplement: Supplementary file 1 [file cancers-18-01897-s001.zip › Supplementary Figures.pdf]

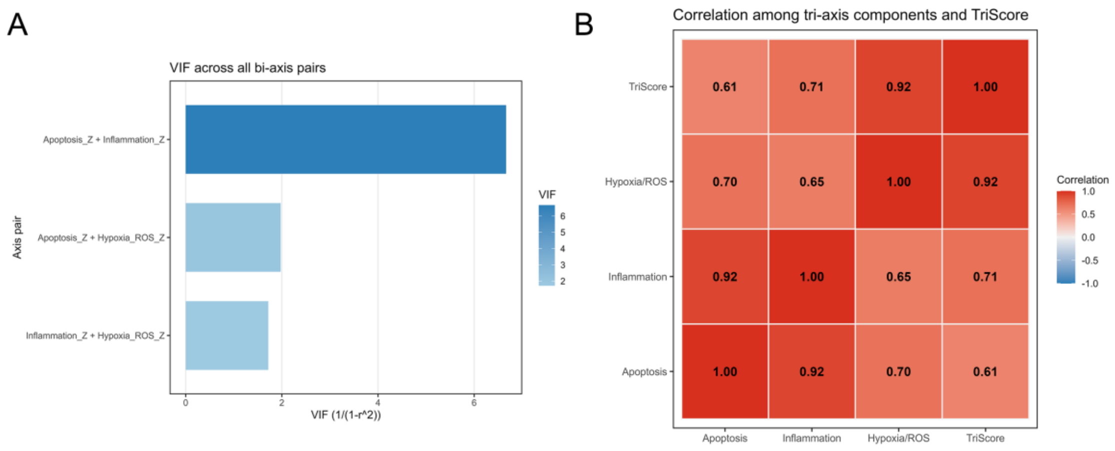

Figure S1. Collinearity and correlation diagnostics for axis selection. (A) Variance inflation factor (VIF) across all bi-axis pairs computed as VIF = $1/(1-r^2)$, where r is the Pearson correlation between axis scores. (B) Correlation heatmap among the three axes (Apoptosis, Inflammation, Hypoxia/ROS) and the harmonized TriScore, illustrating the dependence structure used for model selection.

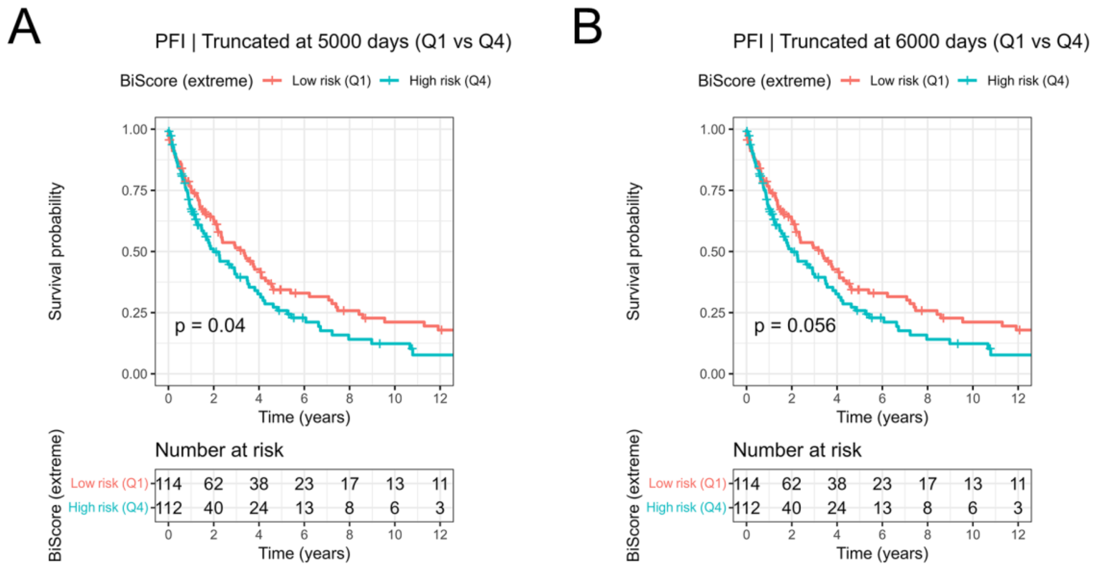

Figure S2. Sensitivity analysis using truncated follow-up. Kaplan–Meier analyses of PFI comparing extreme BiScore groups (Q1 vs Q4) after truncating follow-up time at (A) 5000 days and (B) 6000 days. Time is displayed in years; log-rank P values and numbers at risk are shown.

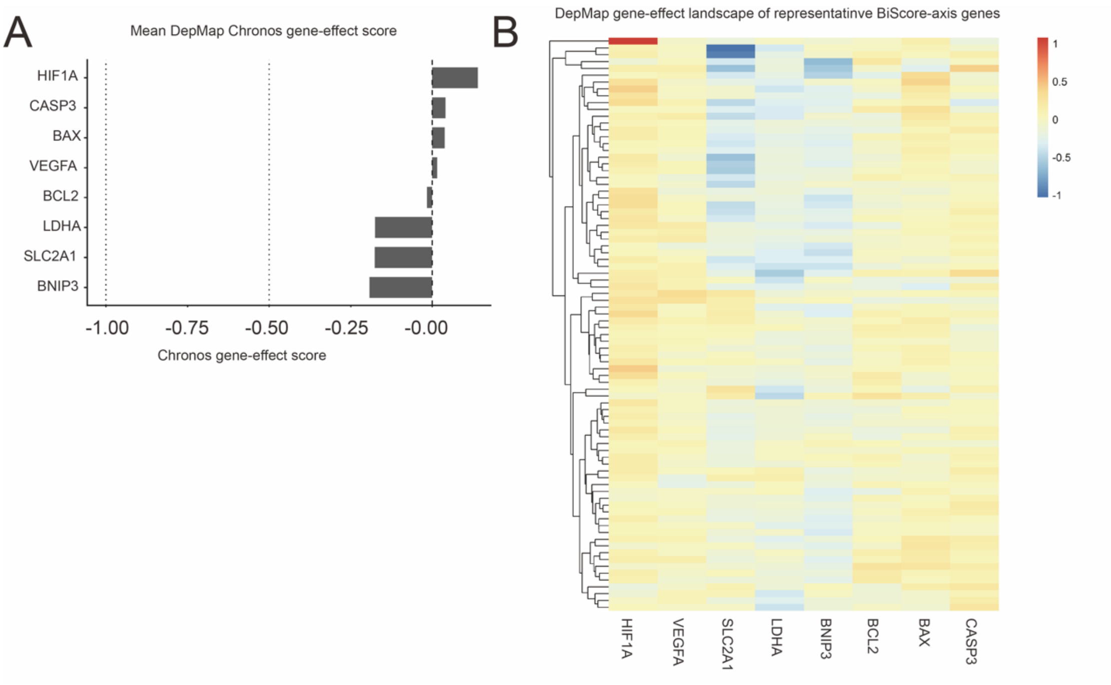

Figure S3. DepMap dependency of representative BiScore-axis genes. (A) Mean Chronos gene-effect scores of selected hypoxia/ROS- and apoptosis-related genes in melanoma cell lines. (B) Heatmap showing heterogeneous gene-effect patterns across melanoma cell lines. More negative scores indicate stronger dependency.
